# Supplementary material for: The genome sequence of the big-headed mining bee, Andrena bucephala (Stephens, 1846)
Source: Wellcome Open Res. 2024 Mar 1;9:111. [Version 1] doi: 10.12688/wellcomeopenres.21003.1 (PMC11322697; doi:10.12688/wellcomeopenres.21003.1)
Supplement: Peer review history

# Reviewer report: Reviewer response for version 1

**DOI:** 10.21956/wellcomeopenres.23238.r90509

**Published:** 2024-08-13

**Authors:** Eduardo Zattara

**Affiliations:** 1 Instituto de Investigaciones en Biodiversidad y Medioambiente, CONICET-Universidad Nacional del Comahue, Bariloche, Argentina

This short and concise data note presents the genome assembly, annotation and preliminary content analysis for a species of mining bee (Andrenidae). It is clearly written, and adequately justifies the rationale for generating the data, and describes the currently standard benchmarks for assembly quality and content. The data are valuable and should be instrumental both in comparative studies of bee genomes and research on this specific species of bee.

 My only observation is that the authors might want to deposit an annotated version of the specific scripts and commands used throughout their assembly and analytical pipelines, and also during the curation process, both to allow for full replicability of the study, but also (and perhaps, more important) to provide other researchers with a template they can use to analyse their own datasets. Such custom code is often hosted at sites like Zenodo or GitHub, which ideally allow version control, support updates and documentation.

Are sufficient details of methods and materials provided to allow replication by others?

Partly

Is the rationale for creating the dataset(s) clearly described?

Yes

Are the datasets clearly presented in a useable and accessible format?

Yes

Are the protocols appropriate and is the work technically sound?

Yes

Reviewer Expertise:

Insect Ecological Genomics

I confirm that I have read this submission and believe that I have an appropriate level of expertise to confirm that it is of an acceptable scientific standard.

# Reviewer report: Reviewer response for version 1

**DOI:** 10.21956/wellcomeopenres.23238.r88900

**Published:** 2024-08-01

**Authors:** Paulo Cseri Ricardo

**Affiliations:** 1 Departamento de Genética e Biologia Evolutiva – Instituto de Biociências, Universidade de São Paulo, São Paulo, Brazil

The article presents data on the assembly of the genome of the bee species *Andrena bucephala*, which is widely distributed throughout Europe. The main result is a final assembly comprising 379.8 Mb, distributed across 13 scaffolds, with five scaffolds accounting for 99.93% of the assembled sequence, in addition to a 19.57 Kb mitogenome. The assembly metrics indicate high quality (QV) and completeness (k-mer completeness and BUSCO). The methods employed were appropriate, utilizing HiFi long reads in conjunction with a Hi-C map to achieve chromosome-scale scaffolds. The protocols are well-described or referenced to ensure reproducibility.

 Overall, the manuscript is written clearly and objectively, presenting highly relevant information for research on the evolution of hymenopteran species, particularly bees. I believe it is suitable for indexing in its current format.

Are sufficient details of methods and materials provided to allow replication by others?

Yes

Is the rationale for creating the dataset(s) clearly described?

Yes

Are the datasets clearly presented in a useable and accessible format?

Yes

Are the protocols appropriate and is the work technically sound?

Yes

Reviewer Expertise:

transcriptomics, genomics, and evolution of bee species

I confirm that I have read this submission and believe that I have an appropriate level of expertise to confirm that it is of an acceptable scientific standard.
